# Genetic Diagnostic Evaluation of Trio-Based Whole Exome Sequencing Among Children With Diagnosed or Suspected Autism Spectrum Disorder

**DOI:** 10.3389/fgene.2018.00594

**Published:** 2018-11-30

**Authors:** Xiujuan Du, Xueren Gao, Xin Liu, Lixiao Shen, Kai Wang, Yanjie Fan, Yu Sun, Xiaomei Luo, Huili Liu, Lili Wang, Yu Wang, Zhuwen Gong, Jianguo Wang, Yongguo Yu, Fei Li

**Affiliations:** ^1^Developmental and Behavioral Pediatric Department – Child Primary Care Department, Brain and Behavioral Research Unit of Shanghai Institute for Pediatric Research and MOE Shanghai Key Laboratory for Children’s Environmental Health, Xinhua Hospital, Shanghai Jiao Tong University School of Medicine, Shanghai, China; ^2^Department of Pediatric Endocrinology and Genetics, Shanghai Institute for Pediatric Research, Xinhua Hospital, Shanghai Jiao Tong University School of Medicine, Shanghai, China; ^3^Developmental and Behavioral Pediatric Department – Child Primary Care Department, Xinhua Hospital, Shanghai Jiao Tong University School of Medicine, Shanghai, China; ^4^Shanghai Children’s Medical Center, Shanghai Jiao Tong University School of Medicine, Shanghai, China

**Keywords:** autism spectrum disorder, whole exome sequencing, diagnostic yield, comorbidity, genetic etiology

## Abstract

Autism spectrum disorder (ASD) is a group of clinically and genetically heterogeneous neurodevelopmental disorders. Recent tremendous advances in the whole exome sequencing (WES) enable rapid identification of variants associated with ASD including single nucleotide variations (SNVs) and indels. To further explore genetic etiology of ASD in Chinese children with negative findings of copy number variants (CNVs), we applied WES in 80 simplex families with a single affected offspring with ASD or suspected ASD, and validated variations predicted to be damaging by Sanger sequencing. The results showed that an overall diagnostic yield of 8.8% (9.2% in the group of ASD and 6.7% in the group of suspected ASD) was observed in our cohort. Among patients with diagnosed ASD, developmental delay or intellectual disability (DD/ID) was the most common comorbidity with a diagnostic yield of 13.3%, followed by seizures (50.0%) and craniofacial anomalies (40.0%). All of identified *de novo* SNVs and indels among patients with ASD were loss of function (LOF) variations and were slightly more frequent among female (male vs. female: 7.3% vs. 8.5%). A total of seven presumed causative genes (*CHD8, AFF2, ADNP, POGZ, SHANK3, IL1RAPL1*, and *PTEN*) were identified in this study. In conclusion, WES is an efficient diagnostic tool for diagnosed ASD especially those with negative findings of CNVs and other neurological disorders in clinical practice, enabling early identification of disease related genes and contributing to precision and personalized medicine.

## Introduction

Autism spectrum disorder (ASD) is a group of highly heterogeneous neurodevelopmental disorders affecting 1 in 59 children aged 8 years, with boys four times more likely to be affected than girls (Baio et al., 2018). It’s characterized by impaired reciprocal social interaction and communication, as well as restricted repetitive interests and behaviors (The Lancet, 2010). The symptoms could develop gradually from early childhood, affecting daily functioning and persisting throughout one’s life (Stefanatos, 2008). Given the variety of phenotypes and severity, it’s believed that genetic factors play a key role in the pathogenesis of ASD, in combination with developmental environmental factors (Hofvander et al., 2009; Mattila et al., 2010; Anagnostou et al., 2014).

The clinical and genetic heterogeneity of ASD has proved to be challenging to the diagnostic workup of affected patients. Routine testing for Fragile X syndrome, karyotyping and chromosomal microarray (CMA) have been established as the first-tier tests for patients with ASD for several years, accounting only for about 1–2%, 5%, and 5–10% cases, respectively (Shen et al., 2010; Betancur, 2011; State and Levitt, 2011; Devlin and Scherer, 2012). Certain loci were identified to confer risk for ASD, and 16p11.2, 15q11-q13, and 22q11.2 were the most frequent (Marshall et al., 2008; Weiss et al., 2008; Fernandez et al., 2010; Hogart et al., 2010; Hiroi et al., 2013). In addition, several genes identified by copy number variants (CNVs) screening and target sequencing for candidate genes were related to ASD susceptibility, such as *PTCHD1, NRXN1, NLGN3, SHANK3, SHANK1* and so on (Jamain et al., 2003; Moessner et al., 2007; Kim et al., 2008; Noor et al., 2010; Sato et al., 2012; Dabell et al., 2013). Recent rapidly improved accuracy and decreased cost of whole-exome sequencing (WES) enabled the application among proband-parent trios of ASD in clinical practice, opening the way to the discovery of single nucleotide variations (SNVs) and indels (Sanders et al., 2012; O’Roak et al., 2014). By using WES, ∼ 20.0% patients with sporadic ASD could be identified and this rate even reached to ∼90% because of the highly inbred nature of the Saudi population, making it useful in complementing CMA designed to detect CNVs, and better characterizing the genetic architecture for ASD in simplex families (O’Roak et al., 2011; Yu et al., 2013; Tammimies et al., 2015; Al-Mubarak et al., 2017). However, thus far, there remains a gap in our knowledge of the diagnostic yield of trio-WES among Chinese children with autistic features when CMA is unable to detect risk-related variations, and its impacts on clinical practice.

It is estimated that more than 70% of individuals with ASD have comorbidities including developmental and psychiatric disorders (Hofvander et al., 2009; Kohane et al., 2012). Based on the suspicion that genetic mechanism of children suffered an abnormality of morphogenesis differed from those who did not, Miles et al. (2005) collected data of dysmorphisms among children with ASD. The patients were further divided into two subsets of patients with documented dysmorphology (complex group) and without evident disrupted morphogenesis (essential group). The findings demonstrated that an abnormal karyotype (2.3%) or a clinically recognized syndrome (1.9%) were identified and restricted to complex group. Triggered by this incentive, we classified patients into different subgroups according to clinical manifestations, to explore the utility of WES and better characterize the underlining genetic differences.

In an attempt to expand the genetic spectrum of ASD by identifying novel SNV and indels, and evaluate how well WES could make up the deficiency of CMA in China, trio-based WES was further implemented among 80 children diagnosed as ASD and suspected of having ASD with negative findings of CMA.

## Materials and Methods

### Patients

Data were collected from children visiting the outpatient clinic of Department of Developmental Behavioral Pediatric and Children Healthcare at Xinhua Hospital, Shanghai, China during March to December 2017. Without detection of CNVs related to ASD, a total of 80 unrelated children (aged 4 months to 13 years) with autistic features were enrolled to further complete trio-based whole exome sequencing (WES). All probands did not have neurological disorders (such as cerebral palsy and schizophrenia) or have the known chromosome/genetic disorders (such as trisomy 21 syndrome, trisomy 18 syndrome, trisomy 13 syndrome, Rett syndrome, Fragile X syndrome). Chromosome microarray analysis were applied by using Cyto Scan HD array (Affymetrix, Santa Clara, CA, United States).

Of these children, 65 (55 males and 10 females) were diagnosed as ASD using standard evaluation including Diagnostic and Statistical Manual of Mental Disorders, Fifth Edition (DMS-5), the Autism Diagnostic Observation Schedule (ADOS), Childhood Autism Rating Scale (CARS) and the intellectual assessment by clinicians. By intellectual assessment, the patients with developmental quotient (DQ) < 75 assessed using Gesell development scales, and intelligence quotient (IQ) < 70 assessed using WISC-R or WPPSI (Wechsler Intelligence Scale for children) were diagnosed as developmental delay or intellectual disability (DD/ID). With related evaluation not available, the rest of children exhibiting clinician-reported autistic features were suspected of having ASD. The study was approved by the ethical committee at Xinhua hospital and conducted in accordance with the relevant guidelines and regulations. Written informed consent in accordance with the Declaration of Helsinki was obtained from the patients and parents. And we have been adhered to standard biosecurity and institutional safety procedures in this study.

### DNA Samples, WES, and Bioinformatics Analysis

Peripheral blood leukocytes from 80 children and parents were obtained. Genomic DNA (gDNA) was extracted using Lab-Aid Nucleic Acid (DNA) Isolation Kit (Zeesan, China), according to the manufacturer’s instructions. The preparation of library of WES was completed using xGen Exome research panel v1.0 (Integrated DNA Technologies, Coralville, IA, United States). Sequencing was performed using paired 150-end, 150-cycle chemistry on the Illumina HiSeq 4000 (Illumina, San Diego, CA, United States), according to the manufacturer’s instructions. Burrows-Wheeler Aligner (BWA, version 0.7.10) was used for FASTQ files to mapping reads to the human reference genome (GRCh37/hg19). Base calling, QC analysis and coverage analysis was performed with Picard tools-1.124 and GATK software. Variants were then annotated using SnpEff version 4.2. Stepwise variant filtering are as follows: variants that demonstrated >1% frequency in the population variant databases including 1000 Genomes Project, Exome Variant Server (EVS) and Exome Aggregation Consortium (ExAC) or >5% frequency in our in-house database (based on 150 exome datasets), and intergenic and 3′/5′ untranslated region variants, none splice-related intronic and synonymous variants were filtered, with those located at canonical splice sites excluded.

Combined with clinical manifestation and modes of inheritance, Sanger sequencing and DNA-based paternity testing were performed to validate the putative pathogenic mutation for all family members. Sequencing products were analyzed using an ABI 3730xl DNA Analyzer (Applied Biosystems, Foster City, CA, United States). For DNA-based paternity testing, the Identifiler^TM^ system and the ABI 3730xL DNA Analyzer (Applied Biosystems) were used to perform multiplex polymerase chain reaction (PCR) amplification and genotyping of PCR products with capillary electrophoresis, respectively. Primer sequences used for validation have been showed in Supplementary Table S1.

MutationTaster^1^, SIFT^2^, and PolyPhen-2^3^ were used to assess the effect of variants on protein function. Validated variants were classified as pathogenic, likely pathogenic, variants of uncertain clinical significance (VUS), likely benign and benign, based on standards and guidelines of the American College of Medical Genetics and Genomics (ACMG). Potential causative genetic variants have been deposited in database of LOVD^4^, with associated accession numbers ranging from #0000379121 to #0000379127.

## Results

### Clinical Characteristics of Patients

The clinical characteristics of children with diagnosed ASD and suspected ASD were summarized in Table 1. Among the patients with ASD, there were 23 children younger than 3 years, 30 children from 3 to 6 year of age (diagnostic rate = 16.7%) and 12 children older than 6 years (diagnostic rate = 8.3%) and the male/female ratio was 53/12. 41 out of 60 patients diagnosed as ASD with available behavioral assessments had CARS scores ≧37 (diagnostic rate = 7.3%), suggesting a severe autistic behavior.

**Table 1:** Clinical characteristics of children with ASD and suspected ASD in the current study.

Characteristic	Children with ASD		Children with suspected ASD	
	No.	Diagnostic rate	No.	Diagnostic rate
Age at testing, y				
<3	23	0/23 (0)	7	1/7 (14.3%)
3–6	30	5/30 (16.7%)	5	0/5 (0)
≧6	12	1/12 (8.3%)	3	0/3 (0)
Sex				
Male	53	5/53 (9.4%)	10	1/10 (10.0%)
Female	12	1/12 (8.3%)	5	0/5 (0)
CARS				
30.0–36.5	19	3/19 (15.8 %)	–	–
37.0–60.0	41	3/41 (7.3%)	–	–
Principal phenotypic feature				
DD/ID	45	6/45 (13.3%)	7	1/7 (14.3%)
Gastrointestinal disorders	25	3/25 (12.0%)	1	0/1 (0)
ADHD	8	0/8 (0)	1	0/1 (0)
Sleep disturbances	6	0/6 (0)	–	–
Craniofacial anomalies	5	2/5 (40.0%)	4	0/4 (0)
Short stature	5	2/5 (40.0%)	1	0/1 (0)
Seizures	2	1/2 (50.0%)	2	0/2 (0)
Congenital heart disease	2	0/2 (0)	1	0/1 (0)
Obesity	3	0/3 (0)	–	–
Microcephaly/Macrocephaly	2	0/2 (0)	2	1/2 (50.0%)

*ASD, autism spectrum disorder; CARS, Childhood Autism Rating Scale; DD/ID, developmental delay or intellectual disability; ADHD, attention deficit hyperactivity disorder; –, absent*.

In terms of other clinical phenotypes, DD/ID was the most common comorbidity in both groups of patients with ASD and suspected ASD. A variety of neurological and non-neurological deficits were exhibit among patients with ASD, including DD/ID (*n* = 45, diagnostic rate = 13.3%), seizures (*n* = 2, diagnostic rate = 50.0%), craniofacial anomalies (*n* = 5, diagnostic rate = 40.0%), etc. (Table 1).

### Molecular Genetic Findings of WES

**FIGURE 1:**
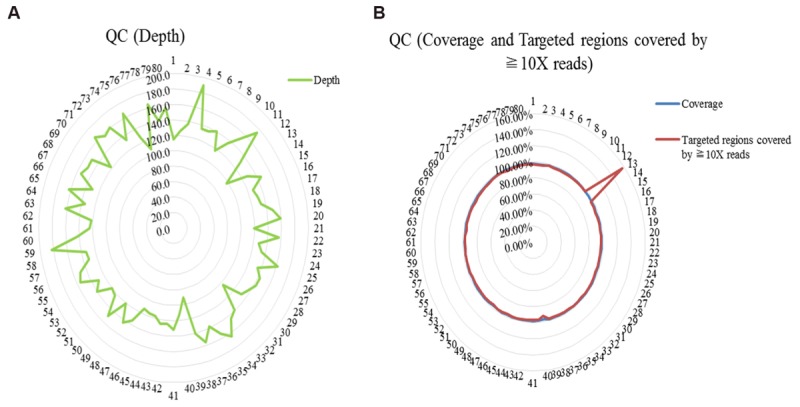
Quality control (QC) of whole exome sequencing. **(A)** QC of the depth; **(B)** QC of the coverage and the targeted regions covered by ≧10X reads.

WES was performed among 80 trios with diagnosed or suspected ASD. Quality control of sequencing showed that 97.8% of the reads were mapped to the reference genome, and 97.7% of the targeted regions were covered by ≧10X reads with enough average depth (138X) (Supplementary Table S2). And details of QC (the depth, the coverage and the target regions covered by ≧10X reads) were shown in Figure 1. Potential causative variants were subsequently confirmed by Sanger sequencing.

A conclusive genetic diagnosis were obtained in seven of 80 children identified by WES, corresponding to an overall diagnostic yield of 8.8% (9.2% in the group of ASD and 6.7% in the group of suspected ASD). We detected and validated a total of seven variants and each was identified in a different gene (Table 2). Based on the distribution of the confirmed variants, frameshift were the most common (3/7), followed by the missense (2/7), stop gained (1/7) and start lost variants (1/7). Among the causative variants, the presumed mode of inheritance was autosomal dominant in 71.4% (71.4% *de novo*), and X-linked in 28.6% (14.3% *de novo* and 14.3% inherited).

**Table 2:** Clinical and molecular findings in children with positive results of WES.

ID	Sex	Age at testing, y	Clinical presentation	Molecular diagnosis	Gene	Sequence variant	Zygosity	Interpretation	ACMG classification
**Diagnosed ASD**									
ASD-685	M	4 years 7 months	DD/ID	Phelan-McDermid syndrome (OMIM:606232)	*SHANK3*	NM_033517.1: c.3630dupG; p.L1210fs	*De novo* het	Frameshift	P
ASD-667	M	4 years 8 months	DD/ID, gastrointestinal disorders	Mental retardation X-linked FRAXE type (OMIM:309548)	*AFF2*	NM_002025.3: c.2509C>T; p.Arg837Cys	Maternal hemi	Missense	P
ASD-706	M	4 years 11 months	DD/ID, seizures, craniofacial anomalies	Mental retardation X-linked 21/34 (OMIM:300143)	*IL1RAPL1*	NM_014271.3: c.1489C>T; p.Arg497^∗^	*De novo* hemi	Stop gained	P
ASD-817	M	7 years 11 months	DD/ID, short stature	White-Sutton syndrome (OMIM:616364)	*POGZ*	NM_015100.3: c.1178_1181 delinsCC; p.H393Pfs^∗^10	*De novo* het	Frameshift	P
ASD-867	F	3 years 2 months	DD/ID	{Autism, susceptibility to, 18} (OMIM:615032)	*CHD8*	NM_001170629.1:c.4611dupA; p.Val1538fs	*De novo* het	Frameshift	P
ASD-821	M	4 years 11 months	DD/ID, craniofacial anomalies, short stature micropenis, anal stenosis,	Helsmoortel-van der Aa syndrome (OMIM:615873)	*ADNP*	NM_015339.3: c.2T>C; p. Met1Thr	*De novo* het	Start lost	P
**Suspected ASD**									
1	M	2 years	DD/ID, Macrocephaly	Macrocephaly/autism syndrome (OMIM:605309)	*PTEN*	NM_000314.6: c.737C>T; p.Pro246Leu	*De novo* het	Missense	P

*ASD, autism spectrum disorder; WES, whole exome sequencing; DD/ID, developmental delay or intellectual disability; P, pathogenic; -, absent*.

*De novo* mutations accounted for 85.7% (six of seven) of the overall molecular diagnoses. Among patients with diagnosed ASD, all of identified *de novo* SNVs and indels were loss of function (LOF) variations and were slightly more frequent among female (male vs. female: 7.3% vs. 8.5%). In addition, all of patients with diagnosed ASD revealed to carry *de novo* LOF variations were co-occurring with DD/ID. A *de novo* missense variation was identified in one patient with suspected ASD. In total, seven genes (*CHD8, AFF2, ADNP, POGZ, SHANK3, IL1RAPL1*, and *PTEN*) with presumed pathogenic variations were identified in this study.

### Impact of WES on Clinical Management

The discovery of WES makes both early clinical detection and genetic counseling possible in various ways among four of seven probands with a conclusive molecular diagnosis. In addition to the following up for ASD based on the risk genes, these patients received further workup of systemic involvement in this cohort. For example, developmental and behavioral evaluation were conducted in the patients with variants of *AFF2* and *IL1RAPL1*, respectively; seizures, short stature, abnormalities of skeleton system, eye, ear, brain and gastrointestinal tract screening, and developmental and behavioral evaluation were implemented in the patient with a *de novo* SNV of *POGZ*; hormone deficiency, short stature, obesity, hypotonia, seizures and feeding problems screening were evaluated in the patient with a *de novo* SNV of *ADNP*. And correspondingly, medication was changed. Growth hormone was applied in patients with *ADNP* and *POGZ* based on the diagnosis of short stature. Brain protein hydrolysate was discontinued in patients with seizures. Two couples with future pregnancy were informed the importance of prenatal testing and preimplantation genetic diagnosis.

## Discussion

With the advent of decreasing cost combined with superior efficiency of WES, studies focusing on the contribution of *de novo* and/or inherited mutations become affordable as well as avoid the potential ‘diagnostic odyssey’ (Bamshad et al., 2011; O’Roak et al., 2011; Tan et al., 2017). In this study, with negative findings of ASD-related CNVs by using CMA, we further confirmed utility of trio-WES for diagnosis among children with ASD or suspected ASD in clinical practice. All *de novo* and inherited variants with predicted damaging effect were validated by Sanger sequencing in both patients and parents. Genetic etiology was identified in seven of 80 trios with an overall detection rate of 8.8%. Within the diagnosed ASD group, six of 65 (9.2%) patients received molecular diagnoses, which was similar to the results observed among sporadic ASD (8.4%), as well as those focusing on either *de novo* or inherited variations, ranging from 6.3% to 13.8% (Neale et al., 2012; Sanders et al., 2012; De Rubeis et al., 2014; Dong et al., 2014; Iossifov et al., 2014; Tammimies et al., 2015). An interesting finding emerging from this study implied the importance of completed ASD-related assessments in enabling a higher diagnostic yield among patients with suspected ASD. Our data showed that compared with children with suspected ASD (6.7%), the diagnostic yield was higher among patients with diagnosed ASD (9.2%). Moreover, diagnostic rate seemed high among ASD patients suffering from other neurodevelopmental disorders including DD/ID, suggesting that patients with these comorbidities may benefit more from WES. These findings were supported by the work of Tammimies et al. who recommended WES as a first-tier test for ASD, especially when comorbid with physical and congenital anomalies (Tammimies et al., 2015). To some extent, the relatively higher yield might result from the patients diagnosed and managed in the outpatient. Those who were diagnosed as ASD especially co-occurring with other neurodevelopmental disorders, are more likely undergone etiological testing including WES, given a high suspicion of genetic etiology.

The past few years have witnessed increasing studies of ASD trios published, highlighting the role of *de novo* variants and improving the identification of candidate risk genes for ASD (O’Roak et al., 2011; Iossifov et al., 2014). Given that *de novo* variation is less frequent and potentially more deleterious, we evaluated its diagnostic rates and effects to determine risk genes. Among children with diagnosed ASD, *de novo* variations were observed in 83.3% of the patients. Moreover, *de novo* LOF mutations contribute to 87.5% cases with ASD. Our findings that *de novo* variations of LOF predominant is contrary to previous population-based studies is intriguing (Iossifov et al., 2012; O’Roak et al., 2012; Sanders et al., 2012). One possible explanation is that ASD patients with other neurodevelopment disorders are prior to be tested by WES in outpatient, and may limit generalizability to the broader ASD population. Another key finding demonstrated here, was that in spite of a predominant male to female ratio (about 4:1), *de novo* LOF mutations were slightly more enriched in females with ASD. And this finding was consistent with the previous results (Levy et al., 2011; Iossifov et al., 2012). Genetic studies suggest that the strong male bias in liability might be attributed to a female protective effect, in which a higher load of mutations were tolerated by female (Gilman et al., 2011; Levy et al., 2011). An increasing body of evidence indicates that affected female with ASD are more susceptible to *de novo* SNVs and indels of LOF (Neale et al., 2012; Sanders et al., 2012; De Rubeis et al., 2014; Dong et al., 2014; Iossifov et al., 2014; Jacquemont et al., 2014). In addition to variations mentioned before, large CNVs encompassing more genes and probably more damaging, are especially abundant in affected females (Levy et al., 2011; Sanders et al., 2011; Jacquemont et al., 2014). These findings suggest to us that other underlying factors that have not yet been identified may contributes much more in males than in females.

All of genes with presumed causative mutations identified here were previously reported in ASD (Herman et al., 2007; Piton et al., 2008; Nishiyama et al., 2009; Bernier et al., 2014; Colak et al., 2014; Helsmoortel et al., 2014; Stessman et al., 2016; Yi et al., 2016). Six genes (*CHD8, AFF2, ADNP, POGZ, SHANK3*, and *IL1RAPL1*) were identified among patients with diagnosed ASD with DD/ID (Piton et al., 2008; Nishiyama et al., 2009; Bernier et al., 2014; Colak et al., 2014; Helsmoortel et al., 2014; Stessman et al., 2016; Yi et al., 2016). In spite of *de novo* LOF variations detected in *SHANK3* and *CHD8*, patient ASD-685 and ASD-867 presented ASD and DD/ID without other disorders at the age of testing. *SHANK3* was a gene encoding a scaffolding protein that is enriched in postsynaptic densities of excitatory synapses (Yi et al., 2016). And *CHD8*, allelic variants of which are associated with ASD, encoding the protein chromodomain helicase DNA binding protein 8 (Nishiyama et al., 2009), which is a chromatin regulator enzyme that is essential during fetal development (Ronan et al., 2013). At present, the mechanism of the higher incidence in males remains inconclusive, and hormones, sex-specific brain differences or variation on the sex chromosomes were speculated to play a role in. We identified two variants (one missense and one *de novo* LOF) in two X-chromosome genes (*AFF2* and *IL1RAPL1*). *AFF2* whose function is to encode a putative transcriptional activator that is a member of the AF4∖FMR2 gene family, was previously associated with ASD and mental retardation, X-linked, FRAXE type (Colak et al., 2014). And in patient ASD-667 with a missense mutation in *AFF2* displayed gastrointestinal disorders in addition to DD/ID. Patient ASD-706 with a *de novo* LOF variation in *IL1RAPL1* showed ASD co-occurring with DD/ID, seizures and craniofacial anomalies. This gene is highly expressed in post-natal brain structures, which functions in the hippocampal memory system, thus suggesting a key role in the physiological processes underlying memory and learning abilities (Gambino et al., 2007). A *de novo* LOF variation in *POGZ* was identified in patient ASD-817 with DD/ID and short stature. Interestingly, previous reports showed patients with variation in *ADNP* often displayed Helsmoortel-van der Aa syndrome (Helsmoortel et al., 2014). However, without obesity and short stature at age of diagnosis, patients ASD-821 harboring a *de novo* LOF mutation (start lost) in *ADNP* presented novel phenotype of micropenis and anal stenosis. After genetic counseling, this patient were screened by biochemical tests related to hormone deficiency, short stature and suggested to be followed up in Department of Pediatric Endocrinology/Genetics. There remains one gene (*PTEN*) with a *de novo* missense variation was detected in a child with suspected ASD. This child presented typically macrocephaly and DD/ID. *PTEN* identified as a tumor suppressor is mutated in a large number of cancers at high frequency (Bonneau and Longy, 2000). These results implied that a continuum of neurological and non-neurological disorders that present in varied patterns might result from candidate risk genes by interacting with other factors.

To our knowledge, this work represents the first comprehensive analysis in Chinese children with diagnosed and suspected ASD by trio-based WES. Similar to other studies by WES, one of potential limitations is that true causative variants may be omitted as a result of stringent criteria to filter false-positives. Besides, WES has limited ability to detect genomic imbalances and could not evaluate variations located on non-coding sequences. Notwithstanding the small sample size, our study in part contributes to dataset of phenotype and genetic etiology of ASD in Chinese children. Moreover, we confirmed the utility of WES in patients without positive results of CNVs, improving the detection rate in a way. Accordingly, many challenges remain, it’s hopeful for a brighter future of individuals with ASD and their families benefiting from the advantages of WES.

## Conclusion

In conclusion, WES offers the advantage of early screening of the underlying ASD-related genes when related CNVs were not identified by CMA, providing genetic diagnoses across diverse clinical subgroups and contributing to precision and personalized medicine.

## Data Availability Statement

The potential causative variants in this study can be found in the LOVD database (https://databases.lovd.nl/shared/variants?search_owned_by_=%3D%22Fei%20Li%22), with associated accession numbers ranging from #0000379121 to #0000379127.

## Author Contributions

FL and YY conceived and designed the study. XG performed the experiments. XD and XG drafted the manuscript. XL and XD collected the samples from ASD families. FL and LS made diagnoses, and interpreted the clinical data. XG, YF, YS, XL, HL, LW, YW, ZG, and JW analyzed the exome sequencing data. XL and KW were responsible for obtaining study ethics and collected clinical data. The authors jointly discussed the experimental results throughout the duration of the study. All authors reviewed and approved the final manuscript.

## Conflict of Interest Statement

The authors declare that the research was conducted in the absence of any commercial or financial relationships that could be construed as a potential conflict of interest.

## Footnotes

1 http://www.mutationtaster.org

2 http://sift.jcvi.org

3 http://genetics.bwh.harvard.edu/pph2/

4 https://databases.lovd.nl/shared/variants?search_owned_by_=%3D%22Fei%20Li%22

## Supplementary Material

TABLE S1 Primer sequences used for validation.

TABLE S2 Quality control of sequencing depth, coverage and the targeted regions covered by ≧10X reads (138X).
